# 2025 List of Reviewers for *OncoImmunology*

**DOI:** 10.1080/2162402X.2026.2651599

**Published:** 2026-04-01

The Editors and Publisher of *OncoImmunology* would like to thank all of our reviewers for their contribution and support during 2025. High-quality peer review is essential to the success of the Journal, and we greatly appreciate the dedication of all those who have contributed to this.

Mohamed Abou-El-Enein

Ibrahim Abukhiran

Joeri Aerts

Ville Äijälä

Yuichi Akama

Mohammad Alhomoud

Maite Alvarez

Beatriz Alvarez-Abril

Lukas Amon

Mads Hald Andersen

Fernando Torres Andon

Luciana Nogueira De Sousa Andrade

Alberto Anel

Marleen Ansems

Angel Arregui

Ilan Bank

Rasha Barakat

Marcus Bauer

Sander Bekeschus

Manuel Beltrán-Visiedo

Nir Ben Chetrit

Fabian Benencia

Pedro Berraondo

Lucillia Bezu

Raymond B Birge

Stéphane Birklé

Moanaro Biswas

Feng Bo

Diana Boraschi

Martin Bornhacurrencyuser

Lucia Borriello

David Boulate

Emilio Bria

Florence Brunet-Possenti

Michael Burger

Claude Capron

Flávia Castro

Giulia Cattaneo

Christophe Caux

Shyambabu Chaurasiya

Xiang Chen

Xiaofei Cheng

Kazuaki Chikamatsu

Bibha Choudhary

Cristina Clement

Gilles Colin

Mario Paolo Colombo

Michael Constantinides

Julie Constanzo

Pio Conti

Zhenyu Dai

Ishani Das

Adil Daud

Maria Davola

Gudrun Debes

Rosa De Groot

Tanja De Gruijl

Mara De Martino

Ingrid A. De Meester

Marc François Diederich

Johannes Doescher

Tiange Dong

Sandra Donnini

Bertrand Dubois

Lelinh Duong

Moshe Elkabets

Elisabeth Eppard

Ahmet Emre EşKazan

Federica Facciotti

Anniina Färkkilä

Camilo Faust Akl

Xiaoming Feng

Carlos Fernández De Larrea

Lucas Ferrari De Andrade

Anna Fialova

Barbara Fingleton

Susetta Finotto

Cinzia Fionda

Silvia Formenti

Katie Freitas

George Fromm

Doriana Fruci

Ezequiel M. Fuentes-Pananá

Shin‑Ichiro Fujii

Udo Gaipl

Julián A. Gajón

Claudia Galassi

Niccolò Gallio

Gina Garcia-Romo

Peter J. Gaskill

Simon Gebremeskel

Markus Gerhard

Olivier Gires

Andrew Godkin

Lígia Catarina Gomes Da Silva

Cécile Gouttefangeas

Juliet Gray

Fabiana Gregucci

Marieke Griffioen

Emma Guilbaud

Nicholas Gulati

Zong Sheng Guo

Niels Halama

Dalil Hannani

Lyna Haybout

Yukai He

Samarth Hegde

Graham Heieis

Kurt Hildebrand

Yoshihiko Hirohashi

Timm Hoeres

Tobias Holderried

Wei-Fan Hsu

Xing Huang

Petra Huehnchen

Yin Pun Hung

Luis Exequiel Ibarra

Hideaki Ijichi

Giorgio Inghirami

Minehiko Inomata

Takamichi Ito

Andrew M Jackson

Shih Sheng Jiang

Songguang Ju

Angel Juarez Flores

Esther Julián

Yuki Kagoya

Abhilasha Kandahalli Venkataranga Nayaka

Oliver Kepp

Jeffrey K. Harrison

Alexander Kirchmair

Kazuma Kiyotani

Astero Klampatsa

Hiroya Kobayashi

Sebastian Kobold

Kathleen Kokolus

Dalu Kong

Takumi Kumai

Julia Kzhyshkowska

Konstantinos Lallas

Justin Durla Lathia

George Lau

Pierre-Antoine Laurent

Henning Lauterbach

Gabrielle Leclercq Cohen

Heung Kyu Lee

Gregory B. Lesinski

Max Levin

Feize Li

Xiaobing Li

Yan-Ruide Li

Zhihao Li

David Liu

Maoxuan Liu

Katharina Lueckerath

Peng Luo

Kewei Ma

Xiaowen Ma

Florencia Madorsky Rowdo

Santos Manes

Michael Marco

Joseph Markowitz

Ana Carolina Martinez-Torres

Inigo Martinez-Zubiaurre

Linxweiler Maximilian

James Mcauliffe

Alexa Simon Meara

Leonid S. Metelitsa

Lydia Meziani

Irene M. Min

Michele Mondini

Carolina L. Montes

Nicolás Montesdeoca

Olivier Morales

Silvana Morello

Kenji Morimoto

Daisuke Muraoka

Shintaro Narita

Jonas Nilsson

Paola Nisticò

Takayuki Ohkuri

Timothy O'Sullivan

Takayo Ota

Erman Ozturk

Changwei Peng

Elísabeth Pérez Ruiz

Federica Perillo

Federico Pietrocola

Antonio Pinto

Kamil Piska

Jonathan Pol

Wen-Bin Qian

Tengyang Qiu

Judith Ramage

Håkon Reikvam

Milan Reinis

Alessandra Rossi

Adhiraj Roy

Michael Rueckert

Dipongkor Saha

Flavio Salazar-Onfray

Sharmila Sambanthamoorthy

Diego Sanchez Martinez

Laura Sanz

Natalia Savelyeva

Nicole Schmitt

Simon Sedej

Barbara Seliger

Sajal Sen

Mahmoud Singer

Kellie N. Smith

Daniel Smrz

Ruth Soler-Agesta

H. Trent Spencer

Mara Steinkamp

Kofi Stevens

Walter J. Storkus

Paola Storti

Zhi-Jun Sun

Dhananjay Suresh

Rei Suzuki

Ghazaleh Tabatabei

Yuichi Tambo

Giuseppe Tarantino

Alvaro Teijeira

Ai-Ping Tong

Jimena Tosello Boari

Tomohide Tsukahara

Sandra Tuyaerts

Stefano Ugel

Alfonso Urbanucci

Luciana Valvano

Claire Vanpouille-Box

Monika Vashisht

Juha Väyrynen

Mitchell Von Itzstein

Derek Wainwright

Jieti Wang

Jing Wang

Na Wang

Xinhui Wang

Youzhi Wang

Emily Webb

Tonya Webb

Feng Wei

Liang Wenhua

Nathan C. Winn

Ai-Wen Wu

Junhua Wu

Shang-Ju Wu

Yijun Wu

Yunong Xie

Shengshan Xu

Showket Yahya

Kimihiro Yamashita

Haitang Yang

Jinlong Yin

Howard Young

Jiali Yu

Kevin Zarrabi

Bei Zhang

Mo Zhang

Yanqiao Zhang

Mingfeng Zhao

Liangxue Zhou

Jon Zugazagoitia

We also thank all our other reviewers who contributed their time and support to the journal, but who requested that we not include their names in this list.

